# Improving Radiology Request Form Compliance: A Clinical Audit at Prince Othman Digna Teaching Hospital, Sudan

**DOI:** 10.7759/cureus.80414

**Published:** 2025-03-11

**Authors:** Abubakr Muhammed, Ali Bashir Mohammed Ahmed, Mohmed H Ahmed Mohmed, Mohamed Elfatih Ali Abdelrahman, Ayman Adil Eltayeb Abdelnour, Mihad Mutasim, Fatima Elsamani Eltayeb MohamedAhmed, Faris Jamalaldeen Mohammed Hamed, Mohammed Abdalla, Moazer Ibrahim Hamid Mohammed, Hayfa Fawzi Ahmed Fadail, Mahir Nourain Mohamed Khiralla, Anwar Musa Abaker Nour, Romisaa Elamin, Mie Saeed, Mustafa H Mohamed, Mohammad Alzain Adam

**Affiliations:** 1 Surgery, University of Gezira, Wad Medani, SDN; 2 General Surgery, Prince Othman Digna Teaching Hospital, Port Sudan, SDN; 3 Medicine, University of Gezira, Wad Medani, SDN; 4 Internal Medicine, Prince Othman Digna Teaching Hospital, Port Sudan, SDN; 5 Internal Medicine, Wad Medani Teaching Hospital, Wad Medani, SDN; 6 General Surgery, Dongola Teaching Hospital, Dongola, SDN; 7 Surgery, Al-Neelain University, Khartoum, SDN; 8 Orthopaedic Surgery, Prince Othman Digna Teaching Hospital, Port Sudan, SDN; 9 Radiology, Prince Othman Digna Teaching Hospital, Port Sudan, SDN

**Keywords:** clinical audit, compliance, patient care, quality improvement, radiology request forms, sudan

## Abstract

Background

Clinical audits serve as a critical tool for quality improvement, evaluating current practices to enhance patient care. Radiology request forms (RRFs) are essential for accurate diagnosis and treatment but often face challenges like incomplete or inaccurate information, illegibility, and non-compliance with established guidelines. This audit aimed to assess the adequacy of RRF completion at Othman Digna Teaching Hospital, Sudan, and implement interventions to improve compliance with guidelines.

Materials and methods

A prospective audit was conducted between November and December 2024. The first cycle involved assessing 50 randomly selected RRFs for adherence to the Royal College of Radiologists (RCR) guidelines. Interventions included introducing standardized forms and providing physician training. A second cycle of 50 RRFs was evaluated post-intervention. Data were analyzed using descriptive statistics and graphical representations.

Results

Compliance with eight key standards improved significantly post-intervention. Patient name compliance increased from 98% to 100%, while patient age rose from 12% to 98%. The inclusion of clinical background details and the question to be answered both reached 100% from initial rates of 16% and 4%, respectively. Overall mean compliance improved from 26% to 95.5%.

Conclusions

The study demonstrated significant improvements in the completion of RRFs following targeted interventions. Regular audits, standardized procedures, and continuous training are essential to sustaining compliance and improving communication between clinicians and radiologists, ultimately enhancing patient care.

## Introduction

Clinical audit is a quality enhancement instrument utilized to evaluate present practices by monitoring, assessing, and improving the quality of patient care. The approach entails assessing an outcome or process, juxtaposing it with existing evidence or optimal practices, and subsequently enacting modifications to enhance the quality of care [1].

Radiology request forms face numerous challenges that impact both patient care and the effectiveness of radiological services. A significant challenge lies in the lack of complete information, as absent details like a patient's comprehensive history or particular areas of concern can influence the precision of image interpretation, possibly resulting in misdiagnoses or necessitating additional imaging procedures. Moreover, the inaccuracies in the information given pose another considerable issue. Mistakes in identifying patients or the chosen imaging method can lead to improper procedures, wasted resources, and delays in treatment. Moreover, the difficulty in reading handwritten forms exacerbates these issues by heightening the chances of misinterpretation and leading to additional delays [2].

The Royal College of Radiologists (RCR) advises that this request form must be filled out thoroughly and clearly to prevent any potential misinterpretation [2]. It is essential for the radiologist to receive adequate and precise clinical information to comprehend the particular diagnostic or clinical issue that the radiological examination aims to resolve, along with a clear articulation of the justifications for the request [3].

When comparing the American College of Radiology (ACR) and the Royal College of Radiology, it becomes clear that a radiology request form needs to include the following details: the patient's clinical history, the question that needs answering, their name, age, address, and phone number; the ward; the requesting doctor's name and signature; the name of the consultant responsible for the patient's care; and the date [3,4]. The radiologist may use the contact information on this form to get in touch with the patient and the doctor who referred them if they need any more information. As a result, it facilitates better dialogue between radiology and other fields. In this manner, reports may be made quickly and efficiently [5].

Systemic analysis was done across Africa, which stated that there is prevalent negligence and inadequate detail in completing radiology request forms, and also a variable degree of non-compliance was observed across all fields [6].

## Materials and methods

Study design and setting 

This prospective audit sought to assess and enhance the radiology request form (RRF) procedure at Othman Digna Teaching Hospital in Sudan, a prominent medical institution catering to a wide range of patients. The audit, which took place from November to December 2024, involved a thorough review of 100 randomly chosen RRFs from different hospital departments. 

Pre-intervention assessment 

The first phase of the audit focused on a comprehensive analysis of the current radiology request procedure. A random sample of fifty RRFs from the hospital's records was examined, uncovering substantial flaws and irregularities in the process. The results pointed out several key areas in need of enhancement, such as the absence of uniformity in the request forms, insufficient clinical details, and a lack of proper training for physicians on how to accurately complete the forms. 

Root cause analysis 

A root cause analysis was conducted to determine the fundamental factors leading to the identified shortcomings. Several key issues were identified, including the disorganization of the request forms, which lacked structure and standardization, leading to confusion and errors. There was also a lack of uniformity across departments, with no consistent approach to completing and submitting RRFs, resulting in variations in request quality and content. Additionally, inadequate physician education and training contributed to many physicians being unaware of the importance of correctly filling out RRFs, which led to incomplete or vague information. Misdiagnosis and diagnostic uncertainty were also prevalent, as unclear clinical notes from physicians often led to errors or ambiguity in diagnosis, resulting in unnecessary retesting and delays in patient management. This not only strained healthcare resources but also caused increased patient discomfort. 

Intervention strategy 

In order to resolve the identified problems, a structured intervention was introduced (Appendix A). The unstructured paper-based RRFs were replaced with a new, standardized template that aligned with the guidelines set by the Royal College of Radiologists (RCR), ensuring consistency and clarity in the request process. The revised RRF was then distributed across different hospital units to standardize the submission procedure and ensure uniform implementation across departments. In addition, a comprehensive training program was launched to educate physicians on the importance of the new format and its correct usage. The program employed a range of approaches, such as delivering PowerPoint presentations at departmental meetings, facilitating group discussions, conducting one-on-one briefings, and using visual displays placed across the hospital. 

Assessment following intervention 

The success of the intervention was evaluated in the second audit cycle, which followed a two-week intervention period. For this review, we used a fresh random sample of 50 RRFs. Focusing on the completeness, correctness, and clarity of the requests, the audit sought to evaluate changes in the radiology request process and compliance with RCR criteria. Using Excel 2016 (Microsoft Corporation, USA), we compared data from the days before and after the intervention. A comprehensive assessment and clear presentation of the findings were facilitated through the use of descriptive statistics. The review included documents such as requests for X-rays, ultrasounds, and echocardiograms. 

Data presentation 

The data collected during both the pre-intervention and post-intervention phases were systematically organized and analyzed to ensure clarity and accuracy in reporting. Descriptive statistics, including frequencies and percentages, were used to summarize the findings related to the completeness, correctness, and clarity of the radiology request forms (RRFs). Bar charts and pie charts were utilized to visually represent trends and comparisons between the pre and post-intervention phases in the hospital audit presentation. These visual aids were created using Excel 2016 (Microsoft Corporation, USA) and were instrumental in highlighting key improvements and persistent challenges in the RRF process. 

Criteria for assessment

Four separate writers checked the RRFs for quality and accuracy by comparing them to the RCR standards. When their evaluations differed, they sought the advice of an impartial third party to settle disagreements and guarantee the audit's validity. 

Ethical clearance 

The Institutional Ethics Committee (IEC) of Othman Digna Teaching Hospital granted approval for this study (IRB number 2024/0154). To safeguard the privacy and confidentiality of the participants, all personal and clinical information was anonymized prior to analysis.

## Results

The compliance with eight key standards was evaluated over two audit cycles, each involving 50 cases, demonstrating substantial improvements across all items. For patient’s name, compliance increased slightly from 98% to 100% (+2%), reflecting consistent accuracy. The patient's age showed significant improvement, rising from 12% to 98% (+86%).

Both the patient's address and the patient's telephone number improved dramatically from 0% in the first cycle to 84% and 82% in the second cycle, respectively (+84% and +82%). The clinical background saw a marked increase from 16% to 100% (+84%), while the question to be answered rose sharply from 4% to 100% (+96%).

The location of the patient improved from 2% to 100% (+98%), and the name of the requesting practitioner increased from 76% to 100% (+24%). These results demonstrate the effectiveness of the interventions in achieving significant and consistent compliance improvements, with many standards reaching full adherence in the second cycle. Table 1 illustrates the compliance in the first and the second cycle.

**Table 1:** The evaluation outcomes and the percentage improvement for radiology request forms.

The standards	First cycle	Second cycle	% of improvement
The patient's name	49 (98%)	50 (100%)	2%
The patient's age	6 (12%)	49 (98%)	86%
The patient's address	0 (0%)	42 (84%)	84%
The patient's telephone number	0 (0%)	41 (82%)	82%
The clinical background	8 (16%)	50 (100%)	84%
The question to be answered	2(4%)	50 (100%)	96%
Location of the patient	1(2%)	50 (100%)	98%
Name of the requesting practitioner	38 (76%)	50 (100%)	24%
Mean compliance	26%	95.5%	69.5%

## Discussion

Since there is minimal opportunity to discuss clinical cases and how both parties manage them, radiology request cards are typically the only way for a clinician and a radiologist to communicate. However, by speaking with the doctor or asking the patient directly, the radiologist or radiographer can get more information. Only when a multidisciplinary approach is taken by the many teams involved in the management can the patient receive the best care possible [7]. It should be noted that incomplete request forms are a global issue.

Our study detected a significant issue with the completion of radiology request forms in the initial cycle, with a compliance rate of only 25%. This low compliance indicates that a substantial portion of the forms were either incomplete or improperly filled, highlighting a systemic issue in the radiology request process at our institution. This finding mirrors the results of Akinola et al., who also identified considerable deficiencies in form completion during their audit [8].

Our study demonstrated that 98% of the radiology request forms included the patient’s name, which reflects a high level of adherence to this critical field. This is a positive outcome, as proper identification is essential for ensuring accurate patient records and avoiding potential errors in diagnosis and treatment. Similarly, Akintomide et al. found that the patient's name was the most consistently filled field in their audit, with a compliance rate of 97.4% [9]. While this is a notable result, several other studies have reported even better completion rates for this field. Specifically, three studies reported compliance rates of 99.85%, 100%, and 100%, respectively [10,11,12]. These findings were achieved in our study in the second cycle, reaching 100% after implementing the interventions, reflecting the importance of these interventions.

In the first cycle, just 12% of forms had the patient's age section correctly filled out, according to our analysis. The second cycle, however, showed a marked increase, with the compliance rate for this sector skyrocketing to 98%. This dramatic improvement is evidence that the therapies carried out between the two cycles were successful. The patient age field was found to be inadequately filled in the research by Akintomide et al. with a compliance rate of 83.45%. Two other trials found compliance rates of 98% and 94.1%, respectively [10,11], so although this was an increase over our first cycle, it was still behind. Nonetheless, it outperformed the results of a single research that found a compliance rate of 79.2% [12]. These comparisons highlight the need for focused interventions to guarantee that this crucial area on radiology request forms is filled out consistently and accurately since the compliance rates across studies are inconsistent.

In our study, the clinical background field was only filled in 16% of the forms in the first cycle, reflecting a significant gap in the completeness of the radiology requests. However, after targeted interventions, this field saw a remarkable improvement, reaching 100% compliance in the second cycle. In contrast, the study by Rajanikanth Rao V revealed that, out of the 200 forms audited, clinical diagnosis was absent in 84 forms (42%), and there were no clinical details provided in 124 forms (62%) [13]. These findings underscore a significant shortcoming in the provision of clinical background information, which is crucial for radiologists to make informed decisions and ensure appropriate imaging. While our study saw a complete turnaround in clinical background completion, the results of Rajanikanth Rao V point to a more pervasive issue that needs to be addressed through consistent training and awareness among referring clinicians.

Comparatively, a similar study conducted by Chukuemeka Agi et al. found that the required clinical information was recorded in 275 out of 300 forms, representing 91.7% compliance [14]. While the Nigerian study demonstrated relatively high compliance, our study saw a complete turnaround, going from a low compliance rate of 16% to full adherence in the second cycle. This highlights the potential for significant improvement through targeted strategies and interventions and underscores the importance of including comprehensive clinical details to support accurate radiological assessments.

In our study, the name of the requesting doctor showed a significant improvement, rising from 76% in the first cycle to 100% in the second cycle, demonstrating a notable increase in compliance. This is comparable to a study conducted in Sudan, where the name of the requesting practitioner improved from 23 forms (46%) in the first cycle to 50 forms (100%) in the second cycle [15]. Similarly, Agi et al. 's study reported that the name of the requesting doctor was filled in 273 out of 300 forms, representing 91.0% compliance [14]. While all three studies show improvements, the compliance in our study and the Sudanese study reached full adherence in the second cycle, highlighting the effectiveness of targeted interventions. In contrast, Agi et al.'s study, while showing high compliance, still leaves room for improvement to reach the same level of consistency observed in our and the Sudanese studies.

The positive impact of improved radiology request form completion on clinical practice cannot be overstated. The accuracy and completeness of the information provided are crucial for effective communication between clinicians and radiologists. When request forms are fully completed, including relevant clinical history and patient details, it enables radiologists to provide more accurate and timely diagnoses, reducing the chances of errors or delays. Moreover, it improves the overall efficiency of the healthcare system by minimizing the need for follow-up clarifications, which can otherwise result in extended waiting times, unnecessary patient visits, or delays in diagnosis and treatment.

In clinical practice, well-completed forms ensure that radiologists have all the necessary information to make informed decisions, contributing to better patient outcomes. The findings from our study suggest that regular audits and interventions to enhance the quality of radiology request forms can lead to more reliable, timely, and effective patient care.

Limitations and recommendations

The study has some limitations, including the relatively small sample size of 50 forms per cycle, which may limit the generalizability of the findings to larger or different populations. Additionally, the research was conducted in a single institution, and thus, the results may not fully reflect practices in other hospitals with different resources or protocols. The study also did not assess which specific interventions were most effective, leaving room for further investigation into targeted strategies. Recommendations for future practice include conducting larger-scale studies with more diverse settings to validate these findings, along with a focus on understanding which interventions have the most impact. Furthermore, continuous training for clinicians and radiologists on the importance of comprehensive and accurate form completion is essential, alongside regular audits to maintain and improve compliance.

## Conclusions

In conclusion, this study emphasizes the critical role that complete and accurate radiology request forms play in facilitating effective communication between clinicians and radiologists. The marked improvements observed after targeted interventions underscore the potential for enhancing clinical practice through regular audits and process changes. While this study achieved significant progress in key areas, such as patient identification and clinical background documentation, it also highlights ongoing challenges in ensuring consistent form completion. The comparison with other studies reveals that while improvements are possible, further efforts are needed to ensure comprehensive documentation across all fields of the radiology request forms. Regular audits, continued clinician training, and the implementation of standardized procedures will be essential in advancing the quality of radiology requests, ultimately contributing to improved patient care and more efficient clinical outcomes.

## Figures and Tables

**Figure 1:**
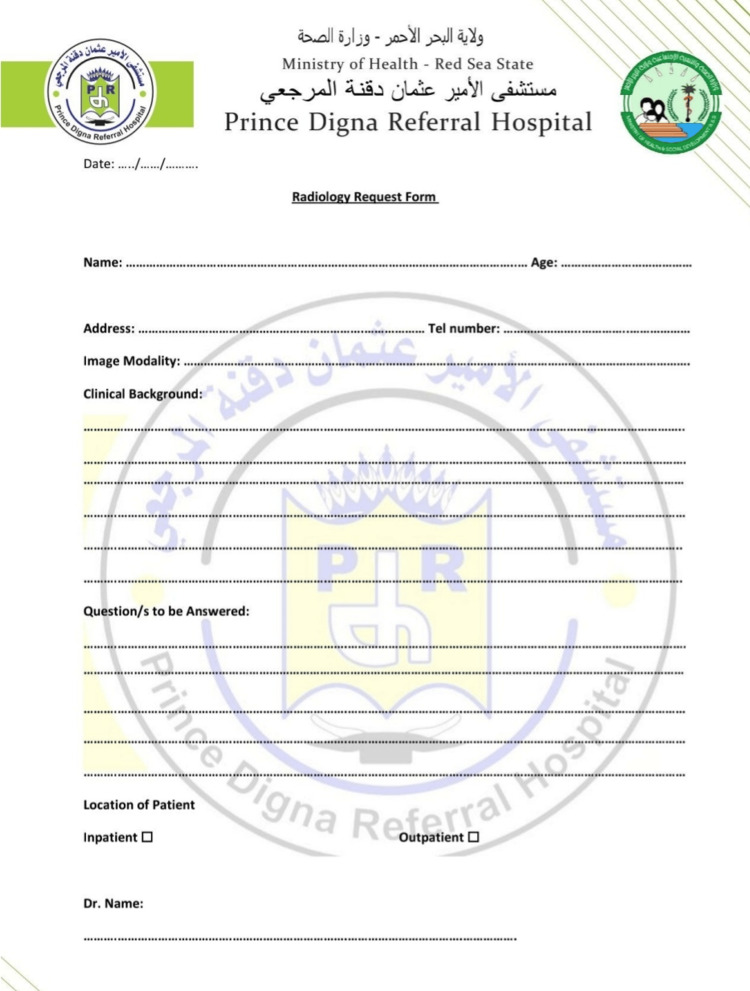
The new radiology request form.

## Appendices

Appendix A 
